# Epidemiology of and Impact of Insecticide Spraying on Chagas Disease in Communities in the Bolivian Chaco

**DOI:** 10.1371/journal.pntd.0002358

**Published:** 2013-08-01

**Authors:** Aaron M. Samuels, Eva H. Clark, Gerson Galdos-Cardenas, Ryan E. Wiegand, Lisbeth Ferrufino, Silvio Menacho, Jose Gil, Jennifer Spicer, Julia Budde, Michael Z. Levy, Ricardo W. Bozo, Robert H. Gilman, Caryn Bern, the Working Group on Chagas Disease in Bolivia and Peru

**Affiliations:** 1 Centers for Disease Control and Prevention, Atlanta, Georgia, United States of America; 2 University of Alabama School of Medicine, Birmingham, Alabama, United States of America; 3 Johns Hopkins Bloomberg School of Public Health, Baltimore, Maryland, United States of America; 4 Hospital Universitario Japones, Santa Cruz, Bolivia; 5 Centro de Salud, Eiti, Gutierrez, Bolivia; 6 Instituto de Investigaciones en Enfermedades Tropicales, Universidad Nacional de Salta, Salta, Argentina; 7 Emory University School of Medicine, Atlanta, Georgia, United States of America; 8 University of Pennsylvania, Philadelphia, Pennsylvania United States of America; 9 Hospital Municipal Camiri, Camiri, Bolivia; 10 Global Health Sciences, University of California San Francisco, San Francisco, California, United States of America; Liverpool School of Tropical Medicine, United Kingdom

## Abstract

**Background:**

Chagas disease control campaigns relying upon residual insecticide spraying have been successful in many Southern American countries. However, in some areas, rapid reinfestation and recrudescence of transmission have occurred.

**Methodology/Principal Findings:**

We conducted a cross-sectional survey in the Bolivian Chaco to evaluate prevalence of and risk factors for *T. cruzi* infection 11 years after two rounds of blanket insecticide application. We used a cubic B-spline model to estimate change in force of infection over time based on age-specific seroprevalence data. Overall *T. cruzi* seroprevalence was 51.7%. The prevalence was 19.8% among children 2–15, 72.7% among those 15–30 and 97.1% among participants older than 30 years. Based on the model, the estimated annual force of infection was 4.3% over the two years before the first blanket spray in 2000 and fell to 0.4% for 2001–2002. The estimated annual force of infection for 2004–2005, the 2 year period following the second blanket spray, was 4.6%. However, the 95% bootstrap confidence intervals overlap for all of these estimates. In a multivariable model, only sleeping in a structure with cracks in the walls (aOR = 2.35; 95% CI = 1.15–4.78), age and village of residence were associated with infection.

**Conclusions/Significance:**

As in other areas in the Chaco, we found an extremely high prevalence of Chagas disease. Despite evidence that blanket insecticide application in 2000 may have decreased the force of infection, active transmission is ongoing. Continued spraying vigilance, infestation surveillance, and systematic household improvements are necessary to disrupt and sustain interruption of infection transmission.

## Author Summary

Despite significant gains in the reduction of the burden of Chagas disease in many South American countries, active transmission and significant burden remain in areas such as the Gran Chaco. High initial vector density, poor housing material, peridomestic infestation, insecticide resistance, and a lack of systematic insecticide spraying and vector surveillance have previously been incriminated for failure to interrupt and sustain interruption of transmission. We conducted a census, seroprevalence, and epidemiologic study in a rural area in the Bolivian Chaco. The prevalence of infection was almost 20% in children and over 80% in adults. We estimated the intensity of transmission over time based on infection prevalence by age. We found that after the first spray program, transmission appeared to fall transiently but then increased again quickly. Sleeping in a structure with cracks in the walls, age and village of residence were associated with increased likelihood of infection. These findings suggest that consistently repeated systematic spraying campaigns accompanied by housing improvements are necessary to interrupt and sustain interruption of vector-borne transmission.

## Introduction

An estimated 8–10 million persons in Latin America are infected with *Trypanosoma cruzi*, the parasite responsible for Chagas disease [1], [2]. In the Western Hemisphere, Chagas disease is responsible for the highest burden of disability-adjusted life years lost among the neglected tropical diseases [3]. Without antitrypanosomal treatment, infection is life-long. Morbidity results largely from cardiomyopathy, which occurs in 20–30% of infected individuals [1]. *T. cruzi* is transmitted by more than 100 species of hematophagous triatomine insects. The parasite is deposited in the vector's feces during a blood meal and can enter the mammalian host through the bite site or intact mucus membranes.

In the 1960s, Chagas disease control programs were initiated in Brazil, Argentina, Chile and other South American countries. Efforts in southern South America were consolidated in 1991 in the first coordinated regional effort, the Southern Cone Initiative (SCI) [4]. The two pillars of the SCI strategy are prevention of transfusional *T. cruzi* transmission through serological screening of blood donations and domestic vector elimination through residual pyrethroid insecticide application and housing improvement. Insecticide campaigns begin with an attack phase in which all houses and peridomestic structures in an endemic community area are sprayed once or twice, followed by surveillance for residual vector foci and reinfestation with focal spraying of affected houses [5]. While housing improvement programs have been limited, insecticide spray campaigns were widely implemented throughout the region [4], [6]. The SCI has led to a marked decrease in the geographic range of *T. infestans* and interruption of transmission by this vector in Chile, Uruguay, Brazil and parts of Argentina [6]–[8]. In Brazil, the seroprevalence in children, used as a proxy for recent transmission, fell from 7.9% in 1990 to 0.1% in 2008 [9], [10]. However, the Gran Chaco, a 1.3 million km^2^ ecological zone shared among Bolivia, Argentina and Paraguay is an exception to this success story [8], [11]. In this region, reports of rapid reinfestation after spray campaigns, emergence of insecticide resistance and sylvatic *T. infestans* populations challenge the strategy of the SCI [5], [12]–[15]. We conducted an epidemiological study in an area of the Bolivian Chaco with serologic evidence of recent transmission despite control efforts. Our objectives were to describe patterns of and risk factors for *T. cruzi* infection, and to examine age-specific prevalence to estimate force of infection as an indicator of transmission over time through the use of catalytic models.

## Materials and Methods

### Ethics statement

The protocol was approved by the Institutional Review Boards of the Centers for Disease Control and Prevention (Atlanta, GA), Asociación Benéfica Proyectos en Informatica, Salud, Medicina y Agricultura (Lima, Peru) and Hospital Universitario Japones (Santa Cruz, Bolivia). All participants 18 years of age or older provided written informed consent. A parent or guardian provided written informed parental consent for all children aged 2–17 years, and in addition, all children 7–17 years signed a simply-worded assent.

### Study area, population, and insecticide spraying history

The Eiti health sector (19°43′52.4994″S, 63°23′9.4812″W, altitude 800 m) is a catchment area composed of 18 villages with a total estimated population of 8320 persons, located in Gutierrez municipality, Cordillera province, Santa Cruz department. The local ecology is sub-humid dry Chaco [8], [16]. Average temperatures range from 16.0–29.1°C, but drop to 5–10°C between the months of May to September [16]. Average annual rainfall is 860 cm, with a rainy season from November to April. The population is almost exclusively of the Native American Guaraní ethnicity, and the local economy is based on subsistence farming and animal husbandry. Houses were predominantly constructed of mud and sticks (*tabique*) or adobe, with packed dirt floors, and straw or corrugated metal roofs; each household comprised 1 to 4 separate structures, usually of one or two rooms. There was no wired electricity and no sewage system. The major water sources were small ponds (*atajados*) in which animals also bathe and drink, or shared or private outdoor taps.

The study area, the Eiti health sector, was chosen based on Ministry of Health Chagas control program data suggesting high likelihood of active *T. cruzi* transmission, including infection prevalence >15% in a sample of children tested in 2006 and high rates of household vector infestation (personal communication, N. Suarez and R. Vargas, Santa Cruz Chagas Disease Control Program, Ministry of Health, 2011). Within the Eiti health sector, we performed a census of seven neighboring communities chosen non-randomly based on size, relative lack of previous interventions, and proximity to our laboratory. The first systematic spray campaign targeting domestic *T. infestans* began in early 2000. Blanket spraying with alphacypermethrin 20% was conducted in 2000 and 2003. From 2005 to 2009, focal spraying of infested houses was conducted; no systematic spraying against triatomines was performed from 2009 to the time of the study. Prior to the 2000 spray campaign, reported household infestation rates ranged from 81.6–100% in the study villages, with an average of 94.3% (based on the reported village level infestation rates weighted by the number of houses in each village). Prior to the 2003 blanket spray, the weighted average infestation rate was 6.9%. Subsequently, the weighted average infestation rates were 3.0%, 18.4% and 57.7% in 2005, 2006 and 2008 respectively.

### Census and epidemiologic data collection

The field team attended community meetings in order to introduce, explain, and answer questions about the study, and to request permission to work in the area. Data were collected from July 2011 to May 2012. Households were defined as all structures that were utilized by a group of people who ate together; the head of household was determined by a consensus of the inhabitants of the household. The target sample size was 2000 based on anticipated *T. cruzi* infection and cardiac morbidity prevalence. All households in the study communities were invited to participate in the census. After obtaining a detailed household roster, the epidemiologic survey was performed, in which the interviewee provided data on the level of education of the head of household, ownership of animals, presence of electricity, and the most recent year in which the house had been sprayed with insecticide. A sleeping structure was defined as a structure in which a person sleeps, irrespective of whether the structure served another purpose. Sleeping structures that share a common entrance were considered a single sleeping structure, while those that did not were considered separate sleeping structures regardless of whether they shared a common wall. For each sleeping structure within a household, observations were recorded regarding the construction material, condition, such as the presence of cracks large enough for vectors to enter, presence of vector fecal traces or exuvia, and traces of lime-based whitewash on the outer walls of the house. Some of the whitewash was provided as part of an intervention program by Caritas, a non-governmental organization, and was sometimes, but not always, accompanied by other housing improvements such as wall plastering and/or substitution of tin roofs for those of thatch.

### Serosurvey

After census and epidemiologic data were collected, trained study nurses visited houses to collect venous blood specimens (5 cc; 3 cc for children 2–5 years old) from all consenting participants 2 years of age or older. Samples were maintained on ice packs and transported within 4 hours to the laboratory of the Hospital Municipal Camiri. Serum and cellular portions were separated by centrifugation, and stored at −20°C until they could be transported to the laboratory at Hospital Universitario Japones in Santa Cruz. All specimens were tested for *T. cruzi* antibodies by the indirect hemagglutination test (IHA; Chagas Polychaco kit, Lemos Laboratories) and Chagatest whole parasite lysate enzyme-linked immunosorbent assay (EIA; Wiener Laboratories, Rosario, Argentina) following the manufacturers' instructions. The IHA was tested in serial dilutions; specimens detectable at 1∶16 dilution were considered to have positive results. Discordant specimens were tested using the Chagatest Recombinante 3.0 ELISA (Wiener Laboratories, Rosario, Argentina). Participants with positive results by at least two assays were considered to have confirmed *T. cruzi* infection [17].

### Statistical analysis

Our analytic approach was to use cross-sectional seroprevalence data to model the population age-specific point seroprevalence using a catalytic model function as described below. After identifying the “best-fit” catalytic model for our data, we applied a force of infection model to this, which we used to estimate the incidence within the population over time (calendar year), taking into account a number of assumptions as described below.

### Census and serosurvey analysis

Serologic test agreement was calculated as the proportion of concordance between the two tests. Differences in seropositivity between the census population and those who participated in the epidemiological survey were tested by chi-square and Wilcoxon rank-sum tests. *T. cruzi* infection prevalence rates by age group were calculated as means, and an age-adjusted prevalence was calculated by applying age-specific rates among those tested to the total population for whom age data were available in the census.

### Catalytic model analysis

We used catalytic modeling from the cross-sectional seroprevalence data to derive age-specific point seroprevalence within our population as a function of age [18]. We tested two parametric catalytic models, constant and Weibull, and a non-parametric cubic B-spline catalytic model to find the best fit to our data [19]. For all models, we assumed (1) no reversion to negative serology (i.e., seropositive individuals will never again be susceptible); (2) the entire seronegative population was susceptible and exposed to the same degree of risk; (3) mortality due to Chagas disease was negligible compared to all-cause mortality and could be ignored; and (4) there was no in- or out-migration other than births and deaths. The constant model also assumed time homogeneity, i.e., that the force of infection remained constant over the study interval, and there were no changes over time in infectivity (infectiousness of the vector and susceptibility to infection of the host remained the same) or transmissibility (including risk factor reduction efforts such as housing improvements) [20]. The Weibull and spline models relaxed the assumption of time homogeneity, as described below.

Model fit statistics and residual plots were used to evaluate which model best fit the data [18]. In the parametric constant and Weibull models, the generated change in prevalence by age cohort is described by a single equation [18]. The constant model has no inflection points within the curve, while the Weibull model had a single inflection point. In the spline model, the participants are divided into multiple cohorts based on consecutive age intervals and the model is fit with a piecewise function using local, smoothed polynomials. The piecewise functions are then connected end-to-end to allow for interval-specific changes in prevalence by age cohorts. The points where the polynomials join are known as knots [21].

Each model was fit to data from all participants for whom age and infection status data were available. In order to test the effect of spray campaigns on transmission, knots were introduced into the B-spline model for the age cohorts whose birth years coincided with the blanket sprays. Additional knots were placed at the minimum and maximum ages, 2 and 91, respectively. We used exploratory data analysis to determine whether and when adding additional knots improved model fit. We used a GLM with a complementary log-log link function in all catalytic models. Thus, our model had the form
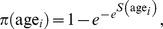
where *π*(age) is the disease prevalence as a function of age, *S*(age) is the linear predictor, and *i* denotes each participant (*i* = 1, …, 1545). With the B-spline basis functions, the linear predictor becomes
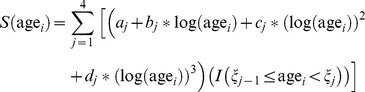
The knots are *ξ*_j_ with boundary knots of *ξ*_0_ = 2 and *ξ*_4_ = 91, the minimum and maximum ages. *I* is an indicator function which equals one if the age falls between *ξ*_j-1_ and *ξ*_j_ and is zero otherwise. Finally, *π*(age) is continuous by forcing *S*(*ξ*_j_) = *S*(*ξ*_j+1_), *S′*(*ξ*_j_) = *S′*(*ξ*_j+1_), and *S″*(*ξ*_j_) = *S″*(*ξ*_j+1_) for the non-boundary knots.

Model fit was assessed by the Akaike information criterion (AIC) [22] using rescaled AIC cutoffs [23] and Pearson's χ^2^ testing, with the cutoff for significance for the latter at p<0.05. The best-fit model was used to determine force of infection. Confidence limits for age-specific seroprevalence were calculated by bootstrap resampling the data 10,000 times with replacement.

### Force of infection model

Force of infection is defined as the proportion of the susceptible population that becomes infected over a given period of time, which we use as an estimate of incidence. In order to calculate this, we used the following function to derive our force of infection, *ℓ*,
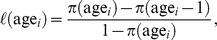
where π(age) is the disease prevalence as a function of age. This function assumes:

1. Uninfected participants are all equally susceptible
2. The difference in prevalence between two adjacent 1-year age cohorts is equivalent to the incidence in the older of the two cohorts during its one additional year of life
3. Disease incidence is independent of age in any given year

The resultant curve estimates the force of infection experienced by the entire susceptible population in by calendar year. Bootstrap 95% confidence intervals were computed for the force of infection at calendar yearby resampling the data 10,000 times with replacement., Any negative incidence estimates as a result of the resampling were truncated at zero [20]. The presented 95% confidence intervals thus represent the set of the linked age-specific confidence intervals. Sample sizes were insufficient to model force of infection at the village level.

### Risk factor analysis

We used Pearson's χ^2^ tests and p-values to test for differences among the variables of interest between villages. To reduce temporal bias between disease acquisition and risk factor presence, we limited the evaluation of associations with risk of *T. cruzi* infection to the subset of our population aged 2–15 years. We constructed logistic regression models with generalized estimating equations to account for clustering at the level of the sleeping structure [24]. Potential risk factors were evaluated in univariable and trivariable models controlling for village and age. Variables to be tested in the full multivariable model (accounting for clustering and all factors in the model) were chosen based on biological plausibility and the change in AIC value [23] and were evaluated for confounding, interaction, and collinearity.

Catalytic and force of infection model analyses were performed in R (R Core Development Team, version 2.13.0, Vienna, Austria). SAS (version 9.3, SAS Institute Inc., Cary, NC) was used for logistic regression models. All statistical tests used the p<0.05 level of significance.

## Results

### Census and serosurvey

Of the 2233 persons recorded during the census, 40 individuals had neither age data nor serum specimens and were excluded from further analyses. Of the remaining 2193 individuals, 107 were younger than 2 years and 541 others declined participation or were absent during multiple house visits. The 1545 serosurvey participants were more likely to be female (54.9% vs 38.5%, p<0.0001) and younger (median age 15 [Interquartile range (IQR) 9–34] vs 20.5 [IQR 12–35], p<0.0001) than the 541 eligible non-participants (Table 1). Participants were more likely to reside in Eiti (30.2% vs 20.7%, p<0.0001) or Paja Colorada (7.6% vs 4.4%, p = 0.01), and less likely to be from Itapicoe (14.7% vs 23.3%, p<0.0001) or El Cruce (17.9% vs 22.3%, p = 0.025) than non-participants.

**Table 1:** Demographic characteristics of participants in the census, *T. cruzi* serosurvey and the risk factor analysis.

Characteristic	Census^1^	Serologic survey^2^	Risk factor analysis^3^
	N = 2193	N = 1545	N = 808
	n (%)	n (%)	n (%)
Male	1087 (49.6)	697 (45.1)	424 (52.5)
Female	1106 (50.4)	848 (54.9)	384 (47.5)
**Median Age (interquartile range)**	15 (8–33)	15 (9–34)	9 (6–12)
**Age distribution**			
≤15 years	1111 (50.7)	808 (52.3)	808 (100)
16–45 years	463 (21.1)	297 (19.2)	
>45 years	619 (28.2)	440 (28.5)	
**Village**			
Eiti	607 (27.7)	466 (30.2)	224 (27.7)
Guasuanti	320 (14.6)	219 (14.2)	119 (14.7)
Sinai	251 (11.5)	184 (11.9)	108 (13.4)
Paja Colorada	154 (7.0)	117 (7.6)	56 (6.9)
Itapicoe	367 (16.7)	227 (14.7)	102 (12.6)
El Cruce	422 (19.2)	277 (17.9)	168 (20.8)
Karavaicho	72 (3.3)	55 (3.6)	31 (3.8)

1 Age data missing for 38 persons.

2 Serological data available for all consenting participants ≥2 years of age.

3 Analytic cohort defined to include all children 2–15 years of age with available serological data.

Of the 1545 individuals tested for *T. cruzi* infection, concordance between IHA and EIA was 95.9%. Concordance between the tests in the subset of 2–15 year old children was 97.0%. The *T. cruzi* seroprevalence among serosurvey participants was 51.7%. The prevalence increased from 19.8% among children 2–15 years old to 72.7% among those 15–30 years old and 97.1% among participants older than 30 years. The age-adjusted prevalence, based on the census population, was 54.1%. The infection prevalence among children 2–15 years old varied significantly by village, ranging from 11.2% in Eiti to 38.7% in Karavaicho (Table 2). There was no difference in prevalence by sex.

**Table 2:** Distribution of potential risk factors among children 2–15 years old by village of residence.

Characteristic	Eiti	Guasuanti	Sinai	Karavaicho	Paja Colorada	Itapicoe	El Cruce	p value^1^
Head of household with education beyond primary school	51/88 (58)	16/38 (42)	17/40 (43)	0/8 (0)	13/19 (68)	26/45 (58)	16/55 (29)	0.0004
House constructed before 2000	34/90 (38)	10/40 (25)	16/39 (41)	2/8 (25)	9/19 (47)	13/46 (28)	18/56 (32)	0.49
Wall construction of mud and sticks	20/90 (22)	19/40 (48)	27/39 (69)	7/8 (88)	6/19 (32)	11/45 (24)	18/56 (32)	<0.0001
Straw roof	2/90 (2)	19/40 (48)	7/39 (18)	6/8 (75)	2/19 (11)	0/45 (0)	10/56 (18)	<0.0001
Unprotected water source	1/89 (1)	2/39 (5)	0/40 (0)	2/8 (25)	0/19 (0)	1/45 (2)	0/55 (0)	0.0002
Propane-generated electricity	7/89 (8)	3/39 (8)	1/40 (3)	0/8 (0)	0/19 (0)	0/44 (0)	0/55 (0)	0.09
Ownership of radio	71/89 (80)	33/39 (85)	31/40 (78)	7/8 (88)	14/19 (74)	40/44 (91)	46/55 (84)	0.6
Report of prior spraying	60/90 (67)	29/40 (73)	28/39 (72)	6/8 (75)	15/19 (79)	31/46 (67)	39/56 (70)	0.95
Whitewash on house	50/90 (56)	16/40 (40)	15/39 (38)	1/8 (13)	4/18 (22)	3/45 (7)	19/55 (35)	<0.0001
Wall cracks	49/89 (55)	31/40 (78)	32/39 (82)	7/7 (100)	11/19 (58)	11/45 (24)	34/56 (61)	<0.0001
Lack of latrine	34/89 (38)	14/39 (36)	22/40 (55)	6/8 (75)	3/19 (16)	18/43 (42)	27/55 (49)	0.03

1 By Chi-square test for the difference among villages.

Several other variables differed significantly by village, including the educational level of the head of household, presence of a protected water source, access to a latrine, and housing materials and conditions (Table 2). In Karavaicho, Sinai and Guasuanti, the majority of houses were built of *tabique* (mud over a stick frame), whereas the majority of houses in other villages were of adobe brick. Traces of whitewash were observed on the walls of 36% of houses overall, but this finding was much more common in some villages (Eiti, Guasuanti, Sinai) than in others (Karavaicho, Paja Colorada, Itapicoe). Cracks in the house walls were much more common in villages where *tabique* houses predominated than in the other villages.

### Catalytic model

**Figure 1:**
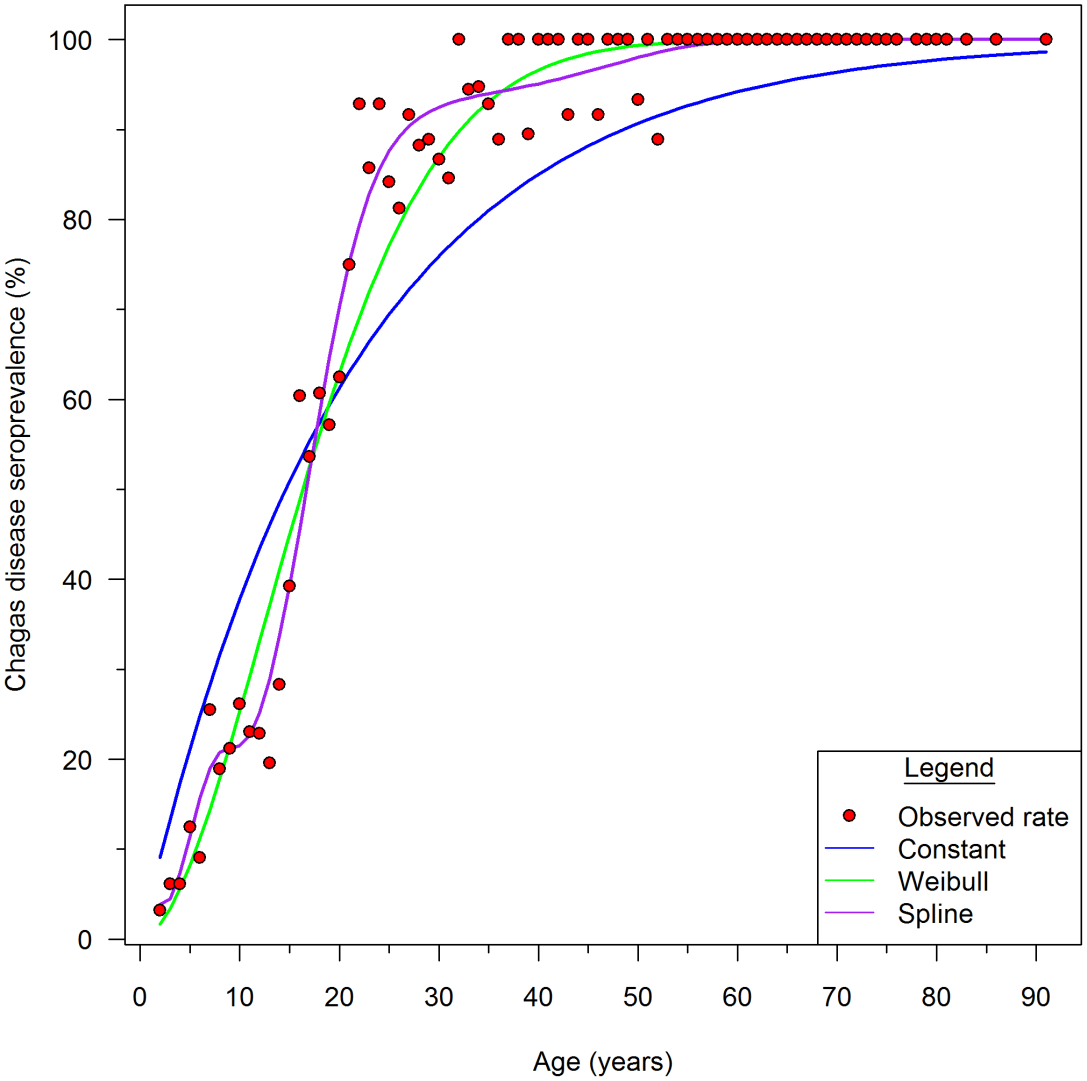
Actual seroprevalence and estimated seroprevalence by the three catalytic models. The scatter plot represents age-specific seroprevalence determined by serologic testing (see methods section). The solid, dashed, and dotted line are estimated seroprevalence curves using the constant, Weibull, and cubic B-spline models, respectively.

**Figure 2:**
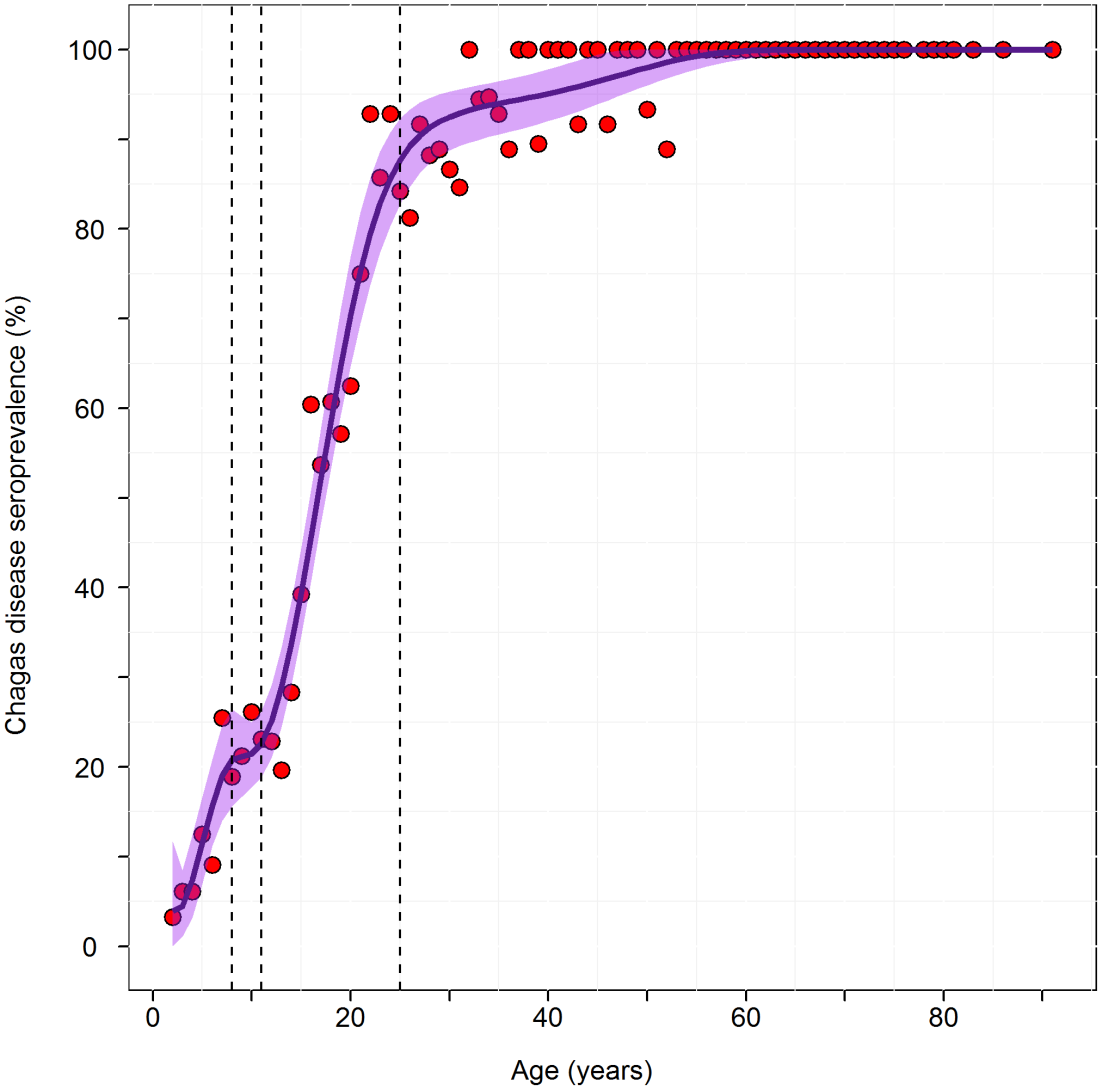
Estimated seroprevalence based on the spline model. Gray shading around the seroprevalence curve represents 95% confidence intervals of model to data points. Vertical dashed lines indicate where knots corresponding to the years of blanket spray campaigns are included in the model.

All three catalytic models demonstrated a steep increase in infection prevalence from age 2 to 30 years; nearly all participants 30 years or older had *T. cruzi* infection (Figure 1). The cubic B-spline catalytic model (AIC = 1212) had better fit statistics than either the constant model (AIC = 1349; χ^2^ = 149.2; df = 6; P = <0.0001) or the Weibull model (AIC = 1227; χ^2 = ^25.1; df = 5; P = 0.0001). The spline model (Figure 2) was therefore used as the basis for the force of infection analysis.

### Force of infection

The force of infection curve was unstable for earlier calendar years due to the small number of susceptible participants in the denominator of the catalytic model, so the analysis focused on the years 1996–2011, corresponding to the birth years of the children aged 2–15 whose data were included in the risk factor analysis and including the blanket spray campaigns in 2000 and 2003. In the exploratory data analysis, we found that an additional knot corresponding to the year of birth of 25-year-olds improved our model fit. Although this knot does not to our knowledge correspond to the year of any intervention, it does suggest a separation of the seroprevalence among persons older than 11 years into one age range (12–24 years) in which prevalence increased with age and another (25 and older) in which nearly all participants were already seropositive.

**Figure 3:**
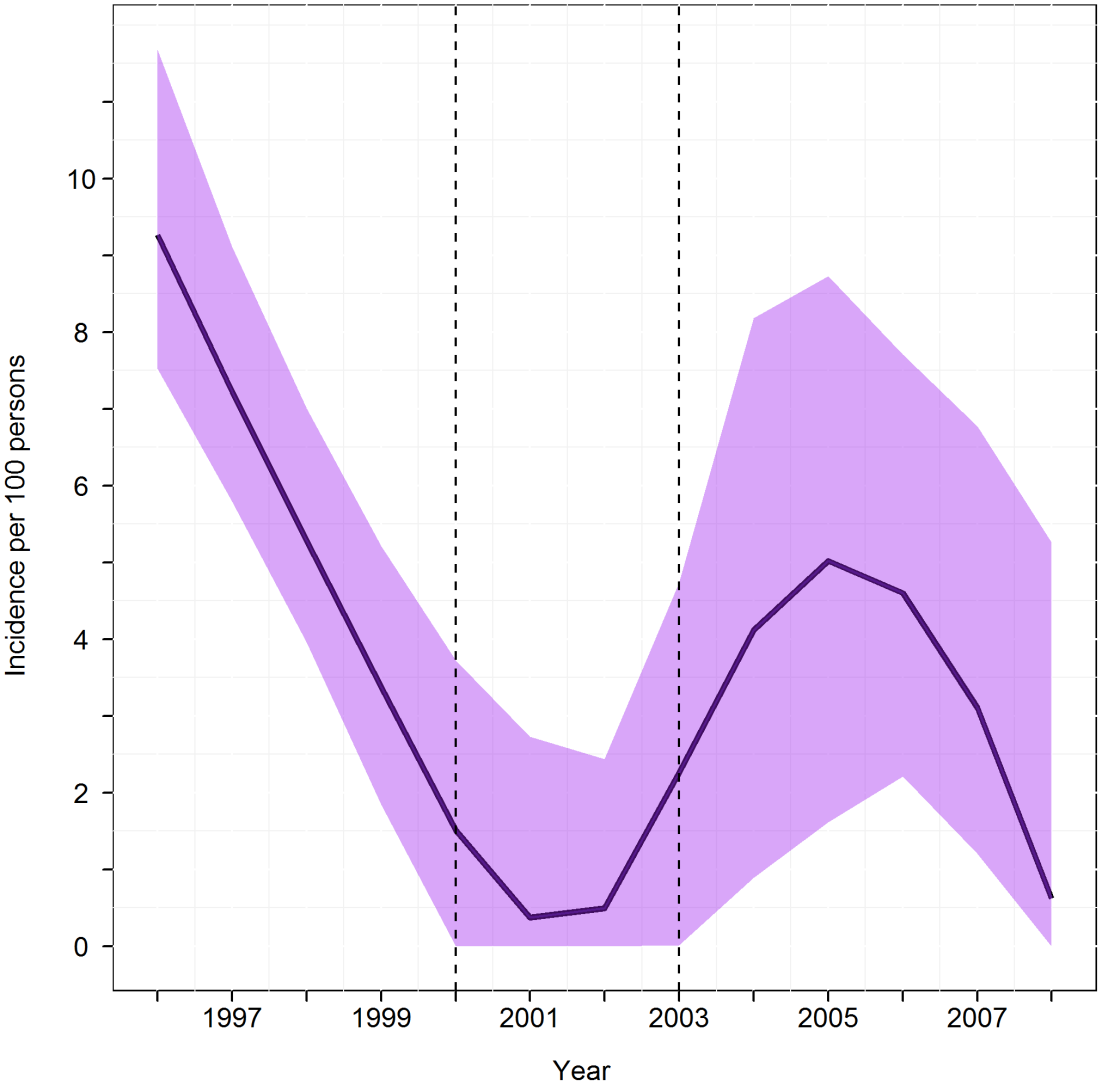
Estimated incidence calculated from the catalytic model. Shaded regions around the line are the 95% confidence intervals of model to data points. Vertical dash lines indicate the knots corresponding to the years of blanket spray campaigns.

The estimated mean force of infection over the time period 1996–2008 was 3.6% per year (Figure 3). Over the two years immediately before the first blanket spray in 2000, the estimated force of infection was 4.3% per year. For the 2 year interval immediately following the first blanket spray, 2001–2002, the estimated force of infection was 0.4% per year. The estimated annual force of infection for 2004–2005, the 2 year period following the second blanket spray, was 4.6%. However, the 95% bootstrap confidence intervals overlap for these estimates, indicating that the differences did not reach statistical significance.

### Risk factors for *T. cruzi* infection among children

In the model adjusted for village, we found a 16% increase in the odds of *T. cruzi* infection with each successive year of life (Table 3). In the age-adjusted model, children from Itapicoe had more than twice the odds, while children in El Cruce and Karavaicho had more than four times the odds of infection compared to those living in Eiti. Because village of residence and age were strongly associated with *T. cruzi* infection, all subsequent analyses were adjusted for these variables.

**Table 3:** Risk factors for *T. cruzi* infection among children 2–15 years old.

	*T. cruzi* infection among those		Unadjusted analyses			Analyses adjusted for age and village		
Risk factor	with factor n/N (%)	without factor n/N (%)	Odds Ratio^1^	95% CI	P value	Odds ratio^1,2^	95% CI	P value
**Age (per year)**			1.15	1.10–1.20	<0.001	1.16	1.11–1.22	<0.001
Female	80/384 (20.8)		1.08	0.79–1.49	0.62	1.12	0.80–1.55	0.51
Male	80/424 (18.9)							
**Village**								
Eiti	25/224 (11.2)		Referent					
Guasuanti	18/119 (15.1)		1.34	0.63–2.85	0.44	1.55	0.73–3.31	0.26
Sinai	16/108 (14.8)		1.46	0.66–3.25	0.35	1.4	0.63–3.12	0.41
Paja Colorada	11/56 (19.6)		2.08	0.81–5.34	0.13	2.21	0.89–5.50	0.09
Itapicoe	21/102 (20.6)		2.13	1.03–4.37	0.04	2.41	1.15–5.06	0.02
El Cruce	57/168 (33.9)		3.98	2.02–7.83	<0.0001	4.42	2.20–8.87	<0.0001
Karavaicho	12/31 (38.7)		5.12	1.86–14.03	0.0015	5.87	1.88–18.31	0.002
**Animal ownership**								
Cats	34/140 (24.3)	117/640 (18.3)	1.32	0.74–2.34	0.35	1.01	0.56–1.84	0.97
Dogs	143/720 (19.9)	8/52 (13.3)	1.43	0.62–3.30	0.41	1.15	0.48–2.79	0.75
Chickens	145/727 (19.9)	6/53 (11.3)	2.73	0.70–10.69	0.15	2.25	0.61–8.31	0.22
**Economic indicators**								
No formal schooling	56/356 (15.7)	94/421 (22.3)	1.52	0.97–2.37	0.07	1.21	0.75–1.94	0. 43
Electricity	2/25 (8.0)	149/751 (19.8)	0.27	0.08–0.89	0.03	0.55	0.17–1.73	0.31
Radio	134/644 (20.8)	17/132 (12.1)	1.76	0.96–3.23	0.07	1.76	0.91–3.40	0.09
**Structure features**								
Reported insecticide spray	119/564 (21.1)	37/221 (16.7)	1.39	0.83–2.32	0.21	1.19	0.71–2.00	0.52
Whitewash on house	44/288 (15.3)	112/497 (22.5)	0.57	0.34–0.93	0.02	0.59	0.34–0.998	0.0495
Stick and mud wall	55/294 (18.7)	101/490 (20.6)	0.96	0.60–1.51	0.85	0.95	0.58–1.56	0.84
Earthen floor	147/742 (18.8)	9/41 (22.0)	0.84	0.37–1.94	0.69	1.11	0.43–2.89	0.83
Metal roof	83/480 (17.3)		Referent					
Tile roof	37/162 (22.8)		1.45	0.86–2.48	0.17	0.92	0.51–1.66	0.78
Straw roof	36/141 (25.5)		1.66	0.92–3.01	0.09	1.34	0.67–2.67	0.41
Cracks in walls	144/676 (21.3)	12/108 (11.1)	1.98	1.03–3.83	0.04	2.16	1.09–4.27	0.03
Gap between wall and roof	102/492 (20.7)	51/234 (17.9)	1.24	0.79–1.94	0.34	1.41	0.87–2.29	0.16
Vector feces or exuvia	127/558 (22.8)	24/193 (12.4)	2.01	1.16–3.49	0.01	1.99	1.15–3.46	0.01

Models included generalized estimating equations to account for correlation at the level of the sleeping structure.

Of 444 sleeping structures in 377 households, 348 structures in 313 households were used by children aged 2–15; serological data were available for 808 children sleeping in 307 structures in 278 households. There was at least one *T. cruzi*-infected child in 75 structures (24.4%) in 72 households. In models adjusted for age and village, sleeping in a structure with traces of whitewash was associated with 40% decrease, whereas observed cracks in the walls and evidence of vector infestation were associated with approximately two-fold increase in the odds of infection. Exploratory analyses suggested that evidence of vector infestation and cracks in the walls were collinear variables. In addition to age and village, only the presence of cracks in the walls remained significantly associated with *T. cruzi* infection in the multivariable model (Table 4).

**Table 4:** Multivariable model of risk factors for T. cruzi infection among children 2–15 years old.

Factor	Odds Ratio^1^	95% CI	p-value
Household radio ownership	1.59	0.81–3.12	0.18
Gaps between the walls and roof	1.35	0.83–2.19	0.23
Cracks in the walls	2.35	1.15–4.78	0.02
Traces of whitewash on house	0.64	0.38–1.08	0.09

1 Adjusted for age and village.

## Discussion

The Gran Chaco has the highest prevalence of Chagas disease in the world [8], [11]. In common with other investigators working in the Bolivian Chaco [25], we found an extraordinarily high *T. cruzi* prevalence, with close to universal infection among adults over 30 years old. Even more disturbing, however, is the fact that nearly 20% of children were infected. Although some study children likely had infection acquired via congenital transmission, the steep rise in prevalence between age 2 and 15 years suggests that vector-borne transmission was responsible for most infections in this age group. Based on our models, the estimated force of infection was above 2.5% per year for most of the past decade, despite major insecticide spray campaigns in 2000 and 2003. Evidence of ongoing transmission in the Chaco poses an urgent challenge to the effort by the Southern Cone Initiative to eliminate *T. cruzi* transmission by domestic *T. infestans* [8], [26].

We used catalytic models to derive the estimated force of infection from age-specific seroprevalence data. Similar models have been used to estimate changes in transmission of *T. cruzi* [27]–[31] and other infections [18], [32]–[34]. The spline model allowed us to include inflection points or knots at the time of the blanket spray programs. The model indicates that transmission was likely already decreasing prior to 2000, possibly as a result of secular changes or activities of local organizations promoting housing improvement. The force of infection dramatically declined in 2000 after the first blanket spray, falling below 1%, but paradoxically increased in each year after the second blanket spray. This suggests that the first blanket spray may have been quite effective, whereas the second blanket spray appears to have had little impact. However, the confidence intervals around these force of infection estimates overlap across all years in this range, suggesting that any impact of blanket spraying was ephemeral. Bolivian Ministry of Health data showed swift reinfestation after the second blanket spray campaign, increasing from 3% of households in 2003 to 58% in 2008 (personal communication, N. Suarez and R. Vargas, Santa Cruz Chagas Disease Control Program, Ministry of Health, 2011), possibly explaining the increasing force of infection estimates over this same time period.

In our study site, several of these factors may have impeded the effectiveness and durable impact of the spray campaigns, including high vector density, vulnerable housing materials, highly infested peridomestic areas, and diminishing resources for intensive surveillance and focal spraying. Although pyrethroid resistance has been reported in *T. infestans* in other parts of the Bolivian Chaco [15], [35], no frank resistance was found in preliminary testing of vectors from our study site (P. Marcet, unpublished data, 2011).

The insecticidal effect of pyrethroids on a house wall is estimated to last from 3 to 9 months, and depends on the type and condition of the wall surface [14]. Nearly all the houses in our study area were of *tabique* (mud over a stick frame) or adobe. Neither is the ideal surface for insecticide application and *tabique* is particularly problematic. Moreover, cracks in the walls of sleeping structures, which provide a refuge for triatomines to avoid contact with insecticide, were associated with an elevated risk of *T. cruzi* in children who slept there. Cracks in the walls were also associated with increased infection risk in children in Argentina and Peru [29], [36]. Studies in diverse sites have demonstrated increased risk of vector infestation and/or human *T. cruzi* infection with adobe or mud walls, and decreased risk with stucco or plastered walls [36]–[39].

In the Paraguayan Chaco, investigators found mortality of *T. infestans* nymphs exposed on mud walls was only 45%, 25% and 0% at 1, 3 and 6 months after insecticide application [14]. A coat of lime was found to increase mortality to 57.5% at 1 and 3 months, but at 6 months, vector mortality was 0% [14]. The improved effectiveness of insecticide applied on lime-painted mud walls may be relevant to our finding that children in whitewashed houses had a lower risk of *T. cruzi* infection. However, the brevity of the entomological effect in the bioassay [14] is consistent with the rapid reinfestation documented in Chagas disease control program data in our study area after 2005. In a study in the Argentine Chaco, a single round of spraying drove infestation rates from close to 100% to undetectable levels, but reinfestation was nearly complete within 5–6 years, attributed to lack of systematic surveillance for reinfestation and inadequate targeted spraying [8], [29], [40]. In that study, the average incidence during a 3-year period beginning four years after the initial blanket spray campaign was 4.3% [29], comparable to our average estimated force of infection of 4.6% in 2004–2006.

Domestic animals, especially chickens and dogs, were found in more than 90% of the houses in our study area. Animal enclosures in the peridomiciliary area are thought to be a frequent source of residual colonies leading to house reinfestation [41]. Peridomestic sites such as chicken coops and pig sties can sustain large vector colonies, are generally of unfinished materials on which insecticide is less effective, and surveillance is insensitive because of the multiplicity of vector refuges [41]–[44]. Mammals may also act as *T. cruzi* reservoir hosts. Dogs appear to be the most important *T. cruzi* infection reservoir in the Argentine study communities mentioned above, with risk for children increasing with the number of infected dogs in the household [29], [45], [46]. In Peru, children who slept in a bedroom with dogs or cats had a significantly increased risk of *T. cruzi* infection compared to those without animals in the bedroom [36]. In our study area, the lack of association between disease status and ownership of dogs or other animals may be due to the ubiquitous ownership of animals, and the fact that dogs freely migrated from household to household, and did not sleep indoors. Data on prevalence of infection in dogs would be useful to further assess their possible role as infection reservoirs.

Age and village of residence were the strongest predictors of *T. cruzi* infection risk in our data. In trivariable models that included these two variables, the only factors that retained statistical significance were traces of vector feces indicating recent or current infestation, cracks in the house walls and whitewash on the house walls. We found no association with socioeconomic indicators or the material of the sleeping structure, factors noted elsewhere to be associated with infection [5], [29], [39], [47], [48]. This may be due to the relative homogeneity in housing structures as many were of vulnerable materials and in poor condition. The strength of association with village of residence suggests that this variable may be acting as a proxy for multiple unmeasured factors.

Our force of infection model requires a number of assumptions. First, the model assumes that all uninfected persons in the population are equally susceptible, implying no difference in immunologic susceptibility or risk factor exposure. Chagas disease, unlike diseases such as malaria which are acquired and cleared repeatedly in persons living in endemic areas, causes lifelong infection and seropositivity without reversion, and so there is no evidence of naturally acquired immunity. The primary risk factor for disease acquisition is sleeping in a structure infested with infected vectors; in our study site housing structures and conditions were fairly similar. The next assumption is that the difference in seroprevalence between two age cohorts separated by one year is the difference in disease acquired by virtue of living that additional year. Finally, the model assumes that disease incidence is independent of age in any year, meaning that the entire population experiences the same force of transmission in a given year. If one already accepts that all uninfected persons are equally susceptible, then it would follow that unless there is a differential in vector affinity to a specific age, that force of transmission is age-independent.

The limitations of our study include the cross-sectional design, which precludes our ability to establish temporality of risk factors with disease acquisition. We cannot say with certainty that a child was sleeping in their current sleeping structure at the time of infection, although in general there was little movement of children between households. As we did not collect systematic data regarding housing improvements other than whitewash, and the organization providing the whitewash often included other interventions for the same houses, we were unable to differentiate the true effect of whitewash versus plastering, roof replacement, focal spraying or other changes. Although we recorded the presence of cracks in the wall large enough for a vector to enter, we did not further differentiate size of cracks. The question of reported insecticide spraying was likely subject to recall bias. Furthermore, comprehensive vector data are not available from the time of the study, impeding a direct assessment of household-level infestation and *T. cruzi* risk.

A large proportion of the population refused to take part in the serosurvey. Those refusing were more likely to be adult males. Thus, as demonstrated by the population adjusted prevalence, the true prevalence in the entire population was likely higher. Refusals in the younger population were much more infrequent. As this was the population from which the risk factor analysis was performed, we expect that refusals played a minimal role in the risk factor analysis. However, due to the age limits imposed upon these analyses we cannot say with any certainty that these same risk factors applied to older populations.

In summary, we found that the study area had an extremely high prevalence of *T. cruzi* infection, with evidence of continued vector-borne transmission despite earlier vector control efforts, and that catalytic modeling provided a useful tool to estimate the force of infection over time and examine the impact of past interventions. As part of a renewed vector control effort in the Bolivian Chaco, municipal teams together with the regional Chagas disease control program performed blanket spraying at the conclusion of our serosurvey. A second round of spraying is planned for 2013. The findings that cracked walls, evidence of vector infestation and whitewashing of walls affected risk of recent *T. cruzi* infection were not unexpected, but highlight the need for systematic housing improvement and effective vector surveillance in the future.

## Supporting Information

Checklist S1 STROBE Checklist.
(DOCX)
